# Absence of Cross-Presenting Cells in the Salivary Gland and Viral Immune Evasion Confine Cytomegalovirus Immune Control to Effector CD4 T Cells

**DOI:** 10.1371/journal.ppat.1002214

**Published:** 2011-08-25

**Authors:** Senta M. Walton, Sanja Mandaric, Nicole Torti, Albert Zimmermann, Hartmut Hengel, Annette Oxenius

**Affiliations:** 1 Institute of Microbiology, ETH Zürich, Zürich, Switzerland; 2 Institute for Virology, Heinrich-Heine-University Düsseldorf , Düsseldorf, Germany; Oregon Health & Science University, United States of America

## Abstract

Horizontal transmission of cytomegaloviruses (CMV) occurs via prolonged excretion from mucosal surfaces. We used murine CMV (MCMV) infection to investigate the mechanisms of immune control in secretory organs. CD4 T cells were crucial to cease MCMV replication in the salivary gland (SG) via direct secretion of IFNγ that initiated antiviral signaling on non-hematopoietic cells. In contrast, CD4 T cell helper functions for CD8 T cells or B cells were dispensable. Despite SG-resident MCMV-specific CD8 T cells being able to produce IFNγ, the absence of MHC class I molecules on infected acinar glandular epithelial cells due to viral immune evasion, and the paucity of cross-presenting antigen presenting cells (APCs) prevented their local activation. Thus, local activation of MCMV-specific T cells is confined to the CD4 subset due to exclusive presentation of MCMV-derived antigens by MHC class II molecules on bystander APCs, resulting in IFNγ secretion interfering with viral replication in cells of non-hematopoietic origin.

## Author Summary

Cytomegaloviruses (CMVs) infect 50 to 90 % of the world's population and cause severe clinical complication in immunosuppressed individuals. An important tissue for horizontal transmission is the salivary gland (SG). CD4 T cells are crucial for viral control in this organ. However, how CD4 T cells control MCMV and why CD8 T cells, important effector cells in other organs, are inefficient in the SG, remains unclear. Here we show that CD4 T cells exert direct antiviral effector rather than helper functions by secretion of IFNγ acting on non-hematopoietic cells. Although SG-resident CD8 T cells were able to produce IFNγ and outnumbered CD4 T cells, absence of MHC class I expression on infected cells due to CMV-encoded immune evasion genes and concomitant absence of cross-presenting antigen presenting cells prohibited antigen recognition by CD8 T cells. Deletion of CMV-encoded immune evasion genes enabled CD8 T cells to control MCMV replication in the SG in absence of CD4 T cells. Hence, CMV control depends on direct antiviral functions of CD4 T cells because of exclusive MHC class II-restricted CMV antigen presentation by bystander APCs in the SG, exemplifying a strategy of effective immune evasion by which CMVs to promote their own transmission.

## Introduction

Cytomegaloviruses (CMVs), members of the β-herpesvirus family, establish a latent persistent infection. Although primary infection in immune-competent individuals is in general clinically silent, severe complications caused by reactivation or primary infection are frequent in immune-compromised patients such as transplant recipients or HIV patients. However, even in individuals with a competent immune system, CMV is detectable in mucosal secretions for a long period after primary encounter, representing the main source for both horizontal and vertical transmission [1]. As sustained replication and shedding of CMVs by the salivary gland (SG) into the saliva is one of the prime reasons for primary and secondary CMV infection (reviewed in [2], [3]), in addition to transmission via breast milk and genital secretions, it is of particular interest for the virus to evade its immune recognition in the SG. Prolonged shedding of CMV into the saliva is also observed in murine CMV infection (MCMV), rendering it a valuable model to identify mechanisms of how CMVs are controlled in the SG [4]. On the host side, specific immune mechanisms are required to control viral replication in the SG: Depletion of CD4 T cells abolished viral control in the SG with sustained viral replication up to 10 weeks post infection [5]. Sustained MCMV replication is restricted in the SG to a particular cell subset, the acinar glandular epithelial cells (AGECs). As systemic neutralization of IFNγ and TNFα abolished antiviral MCMV control in the SG, it was proposed, but never directly proven, that CD4 T cells control viral replication via secretion of IFNγ and TNFα [6], [7]. MCMV-specific CD4 T cells were indeed found to produce both of these cytokines [8], [9], but it remains unclear whether CD4 T cells directly control MCMV replication via secretion of IFNγ and TNFα. Further, although never directly addressed experimentally, it was proposed that IFNγ secreted by virus-specific CD4 T cells may act on other immune cells such as NK cells to induce antiviral activities in these secondary effector cells and not directly on infected target cells [4]. In contrast to CD4 T cells, CD8 T cells and B cells seem to be dispensable for MCMV control in the SG [10], [11], [12].

Many open questions remain as to why CD4 T cells are so crucial to control MCMV in the SG and not - or to a lesser extent – in other tissues. Up to date it is unclear whether CD4 T cells are necessary to support the recruitment or function of other immune cell subsets in the SG during MCMV infection, as immune responses to MCMV have not been investigated comprehensively in this relevant tissue. To investigate in detail local immune control of MCMV replication in the SG, we identified the exact mechanisms of antiviral control exerted by CD4 T cells. We prove that production of IFNγ by CD4 T cells is essential to control MCMV replication and IFNγ needs to be sensed by radio-resistant cells and not by cells of the hematopoietic lineage. Despite being able to secrete IFNγ and outnumbering MCMV-specific CD4 T cells, MCMV-specific CD8 T cells are unable to control MCMV replication in the SG due to extensive virus-induced down-regulation of MHC molecules on infected AGECs. Hence, antigen recognition in the SG depends on local professional APCs having taken up and processed exogenous MCMV-derived antigens. Intriguingly, SG-resident APCs are unable to cross-present particulate antigens, resulting in severe paucity of MHC class I but largely intact MHC class II presentation of MCMV-derived epitopes on these APCs. Thereby MCMV efficiently evades its immune recognition and elimination by CD8 T cells, ensuring prolonged viral shedding into the saliva and promoting horizontal transmission.

## Results

### Helper functions exerted by CD4 T cells are dispensable for control of MCMV infection in the SG

**Figure 1:**
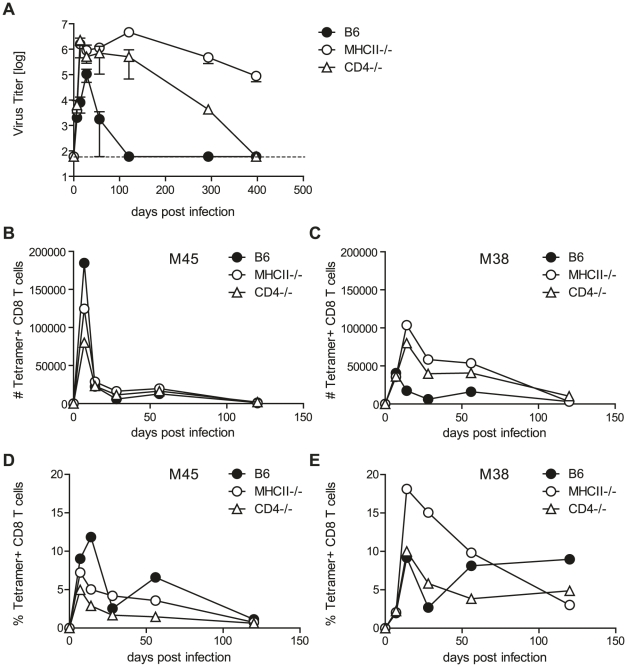
Helper functions exerted by CD4 T cells are dispensable for MCMV control. (A) MCMV titers in the SG in CD4^-/-^ (open triangles), MHCII^-/-^ (open circles) and B6 (closed circles) mice at different time points post infection. Dotted line shows detection limit. (B–E) M45- and M38-specific CD8 T cells were quantified by tetramer staining of pooled SG leukocytes of CD4^-/-^ (open triangles), MHCII^-/-^ (open circles) and B6 (closed circles) mice at different time points post MCMV infection. Representative results of three independent experiments are shown.

CD4 T cells are crucial to control MCMV infection, especially in the SG where productive viral replication continues in the absence of these cells, at least for 10 weeks which was the longest observation period in previous studies [5], [6]. We corroborated this finding and extended the observation period to more than one year, by comparing viral titers of two mouse strains that lack CD4 T cells, namely MHC class II knockout (MHCII^-/-^) and CD4 knockout mice (CD4^-/-^), with wild type C57BL/6 (B6) mice at different stages post MCMV-Δ*m157* (herein after referred to MCMV) infection. *m157* is expressed on the surface of MCMV-infected host cells and interacts with the activating receptor Ly49H on NK cells of B6 mice, thereby leading to enhanced viral control. We deliberately used an *m157* deletion mutant in our studies as most laboratory mouse strains as well as tested outbred mice lack expression of Ly49H [13], [14]. Using plaque forming assays, presence of replicating MCMV was analyzed in several organs including the spleen, lung, liver and SG over more than one year. In the absence of CD4 T cells, increased viral titers and prolonged detection of replicating virus was observed for all organs examined. However, with the striking exception of the SG, infectious virus was eventually controlled in spleen, liver and lung (Fig. S1). In the SG infectious MCMV was still detectable in MHCII^-/-^ mice, even more than one year post infection (Fig. 1A). Surprisingly, in CD4^-/-^ mice, MCMV replication in the SG was eventually controlled between 200 to 400 days post infection.

CD4 T cells can control pathogens by several means, a prominent function being provision of help to either CD8 T cells or B cells which then control pathogen replication via cytotoxicity or production of antibodies. To test if CD4 T cells control MCMV by exerting helper mechanisms, we analyzed virus titers of MCMV infected CD8 T cell (CD8^-/-^) and B cell deficient mice (JHT mice). SG virus titers in both of these mouse strains were comparable to wild type animals at four (Fig. S2A) and eight (Fig. S2B) weeks post infection; at both time points, however, viral titers were markedly increased in MHCII^-/-^ mice. Our results are consistent with previous reports of efficient viral control in the absence of CD8 T cells and B cells [10], [11], [12]. It is possible, however, that MCMV-specific CD8 T cell responses were impaired in the SG of CD4 T deficient animals, leading to impaired viral control. This would be a plausible explanation for the requirement of CD4 T cells to control MCMV replication in the SG which has not been addressed so far. To exclude a role of T cell help for CD8 T cells in the SG, we analyzed CD8 T cell responses in CD4 T cell deficient and control B6 mice (Fig. 1B-E). SG-resident lymphocytes were isolated and CD8 T cells specific for the epitopes M45 (Fig. 1B, D) and M38 (Fig. 1C, E) were quantified by tetramer staining. M45-specific CD8 T cell responses in mice lacking CD4 T cells were largely comparable to wild type mice and M38-specific CD8 T cell responses were generally increased in MHCII^-/-^ and CD4^-/-^ mice, indicating that MCMV-specific CD8 T cell responses in the SG were not impaired in absence of CD4 T cells up to 120 days post infection.

### CD4 T cells control MCMV replication by secretion of IFNγ

**Figure 2:**
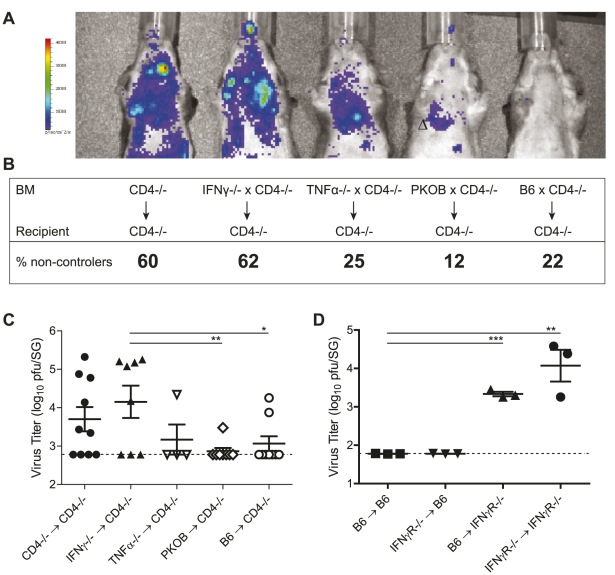
CD4 T cells exert direct antiviral mechanisms by secretion of IFNγ. (A) γ-irradiated CD4^-/-^ were reconstituted with 50% bone marrow of CD4^-/-^ mice and 50% bone marrow of either CD4^-/-^, IFNγ^-/-^, TNFα^-/-^, PKOB or B6 mice. Mice were infected with a MCMV mutant expressing the firefly luciferase under the control of the *m157* promoter. 10 minutes prior to analysis, D-luciferin was injected i.p. and active virus replication was detected by *in vivo* bioluminescence imaging in one month infected chimeras. (B, C) 2 to 4 months post infection viral titers were determined by plaque assay. Dotted line shows detection limit. Combined results of three individual experiments are shown. Percentages of mice bearing a virus load above the detection limit (non-controllers) are indicated in B and the virus titers are shown in C. (D) IFNγR^-/-^ or B6 mice were γ-irradiated and reconstituted with of B6 or IFNγR^-/-^ bone marrow, respectively. MCMV titers in the SG were determined 8 weeks post infection. Representative results of two independent experiments are shown. (* p<0.05, ** p<0.01, *** p<0.001, 2-tailed unpaired student's t-test).

Previous studies indicated that systemic administration of neutralizing antibodies specific for either IFNγ or TNFα could abolish CD4 T cell-mediated control of MCMV replication in adoptive transfer models [6], [7]. Further, in CMV-seropositive humans, direct cytolytic activity exerted by virus-specific CD4 T cells was proposed [15]. Although IFNγ and TNFα are involved in the control mechanisms exerted by CD4 T cells, it remains unknown if CD4 T cells produce these cytokines themselves and which cell type these mediators act upon. To identify the effector molecules produced directly by CD4 T cells to control SG MCMV replication, we generated the following mixed bone marrow chimeras: CD4^-/-^ recipients were γ-irradiated and reconstituted with 50% bone marrow of CD4^-/-^ and 50% bone marrow of either IFNγ deficient (IFNγ^-/-^), TNFα-deficient (TNFα^-/-^), perforin-deficient (PKOB), CD4^-/-^ or B6 mice. By doing so, CD4 T cells present in the reconstituted animals were the only cell subset being entirely deficient for either IFNγ (IFNγ^-/-^xCD4^-/-^), TNFα (TNFα^-/-^xCD4^-/-^) or perforin (PKOBxCD4^-/-^). Control animals lacked CD4 T cells completely (CD4^-/-^xCD4^-/-^) or CD4 T cells were fully functional (B6xCD4^-/-^). At the time point of infection, frequencies of CD4 T cells were comparable in all experimental groups harboring CD4 T cells (data not shown). The chimeric mice were infected with a recombinant MCMV expressing the firefly luciferase under the control of the *m157* promoter [16]. In these animals active viral replication is detectable by *in vivo* bioluminescence imaging after intraperitoneal administration of D-luciferin (Fig. 2A). Furthermore, two to four months post infection MCMV titers were determined in the SG by plaque assay. Percentages of non-controllers (viral titer above detection limit) and the viral titers are shown in Fig. 2B and C. Viral control was impaired in (CD4^-/-^xCD4^-/-^) compared to (B6xCD4^-/-^) chimeras, corroborating our results in non-chimeric mice. While a comparable lack of MCMV control was observed in mice in which CD4 T cells were deficient for IFNγ production or in which CD4 T cells were completely absent, MCMV replication was similarly controlled in the SG of chimeric mice in which CD4 T cells lacked TNFα or perforin and in mice with fully functional CD4 T cells. To exclude impaired CD4 T cell priming in any of the experimental groups, frequencies of lung-derived MCMV-specific CD4 T cells secreting IFNγ or TNFα were analyzed and were comparable in all CD4-sufficient animals (data not shown). These data strongly support the notion that CD4 T cells directly control MCMV replication in the SG by secretion of IFNγ.

Next, we addressed the question whether IFNγ secreted by CD4 T cells inhibited viral replication in the SG by signaling via IFNγ receptors (IFNγR) expressed on non-hematopoietic cells (including infected AGECs) or by triggering IFNγR signaling on hematopoietic cells, thereby stimulating other immune cells such as NK cells to control viral replication. Using either IFNγR^-/-^ or wild type mice as recipients or donors, bone marrow chimeras were generated that either lacked IFNγR completely (IFNγR^-/-^ → IFNγR^-/-^), that lacked IFNγR on radio-resistant cells (B6 → IFNγR^-/-^), that lacked IFNγR on radio-sensitive cells (IFNγR^-/-^ → B6) or that expressed IFNγR on all cells (B6 → B6). 8 weeks post MCMV infection, viral titers increased in the SG when IFNγR was completely absent compared to wild type counterparts (Fig. 2D). Importantly, increased lytic viral replication was only observed in mice which either completely lacked IFNγR expression or selectively on non-hematopoietic cells. In contrast, mice that lacked IFNγR only on hematopoietic cells controlled MCMV replication. 8 weeks post infection, MCMV-specific CD4 T cell responses were comparable between experimental groups (data not shown). Hence, IFNγ secreted by CD4 T cells signaled on radio-resistant cells to suppress viral replication.

### IFNγ-secreting CD8 T cells outnumber IFNγ-producing CD4 T cells in the SG and are abundantly present in the absence of CD4 T cells

**Figure 3:**
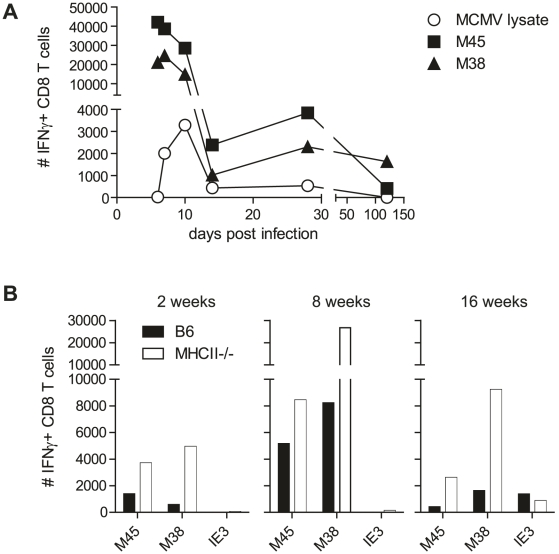
MCMV-specific CD8 T cells isolated from the SG secrete IFNγ. (A) Kinetic analysis of the number of IFNγ-secreting CD8 T cells after *ex vivo* restimulation with the epitopes M45 (closed squares) and M38 (closed triangles) or CD4 T cells after stimulation with a lysate of MCMV infected cells (open circle) in the SG. B) Total numbers of IFNγ-producing CD8 T cells isolated from SG of wild type (black bars) or MHCII^-/-^ (open bars) mice after *ex vivo* restimulation with either M45, M38 or IE3 peptides after 2, 8 or 16 weeks post infection. Data of SGs pooled from three to four mice per group are displayed.

CD8 T cells and NK cells from the spleen were shown to produce IFNγ upon MCMV infection [17], [18]. Why is then exclusively IFNγ produced by CD4 T cells so crucial to control viral replication in the SG? We first tested if IFNγ production by MCMV-specific CD8 T cells isolated from the SG was impaired. Numbers of CD8 T cells secreting IFNγ after *ex vivo* restimulation were much higher compared to MCMV-specific CD4 T cells at any time point examined (Fig. 3A), indicating that MCMV-specific CD8 T cells *per se* were able to produce IFNγ.

CD4 T cells were shown to be necessary for the development of fully functional CD8 T cells in a variety of infection models (reviewed by [19]). Although the absence of CD4 T cells only slightly impacted the functionality of CD8 T cells isolated from the spleen, lung or liver [5], [20], [21], [22], the possibility remained that SG derived MCMV-specific CD8 T cell function was crucially dependent on CD4 T cells. However, in the SG, IFNγ-producing CD8 T cells specific for either the M45 or the M38 epitope were increased rather than decreased in the absence of CD4 T cells at various time points post MCMV infection (Fig. 3B). Even IE3-specific CD8 T cell responses, which have previously been shown in the blood and spleen to largely depend on presence of CD4 T cells [22], were detected to almost comparable levels in the SG in presence or absence of CD4 T cells. These data clearly argue against a role for CD4 T cells to support CD8 T cell function in the SG at the investigated time points.

### CD8 T cells as well as NK cells infiltrate the SG and migrate to infected cells in the absence of CD4 T cells

**Figure 4:**
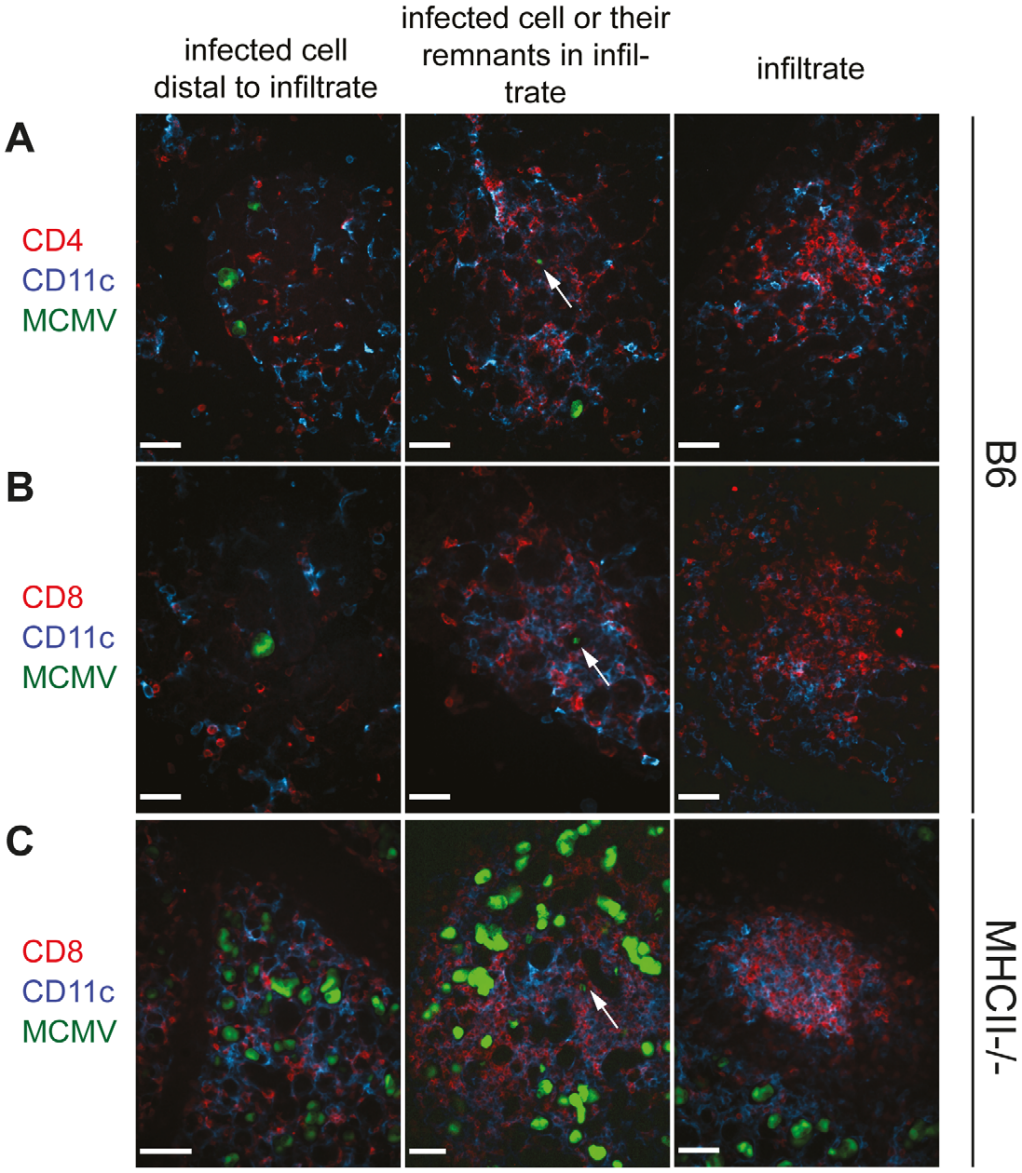
CD4 as well as CD8 T cells infiltrate the infected SG tissue and CD8 T cell infiltration is CD4 T cell independent. SGs were isolated three weeks post infection from B6 (A and B) or MHCII^-/-^ (C) mice infected with a GFP-expressing MCMV mutant. Cryosections of SG were counterstained with anti-CD4 (A; red) or anti-CD8 (B and C; red) and anti-CD11c (blue). MCMV-bearing AGECs (green) were situated either distal to immune infiltrates (left column) or in proximity to infiltrates (middle columns). In some infiltrates cells with very low fluorescent intensity for GFP were present (middle column; arrow), most likely representing cells not directly infected with MCMV but instead cells that had taken up remnants of MCMV-infected cells. Dense leukocyte infiltrates were often devoid of GFP^+^ cells (right column). Confocal images were taken with 20 times magnification. Scale bar indicates 100 µm. One representative picture of a minimum of 10 is shown.

Although MCMV-specific CD8 T cells and NK cells were detectable in the SG tissue by flow cytometric analysis, their migration to close proximity of infected cells might be less efficient in comparison to MCMV-specific CD4 T cells. To determine the homing and tissue distribution of CD8 T cells and NK cells in relation to MCMV-infected cells, we performed confocal immunofluorescence analyses on tissue sections of the SG. B6 and MHCII^-/-^ mice were infected with a GFP-expressing *m157*-deficient MCMV mutant. Three weeks later SG sections were stained for CD4 T cells, CD8 T cells, NK cells, B cells and CD11b as well as CD11c expressing cells (Fig. 4 and Fig. S3). As shown previously [5], [23], AGECs were the main cells of the SG that were infected with MCMV at this time point and displayed cell enlargement typical for MCMV infected cells (Fig. 4). Scattered throughout the SG tissue, dense foci of leukocyte infiltrates were detectable comprising not only CD4 and CD8 T cells but also CD11c and CD11b expressing as well as NK and B cells. However, these dense foci of leukocytes most often did not contain GFP^+^ cells (Fig. 4 and Fig. S3; right columns). We propose that these infiltrates had previously surrounded a GFP-positive MCMV-infected cell that had been eliminated and is hence no longer detectable via GFP expression. GFP-expressing (hence MCMV infected) AGECs were either located distal, proximal or in very rare occasions within leukocyte infiltrates. Only few immune cells were in close proximity to infected cells when situated distal to infiltrates (Figs. 4A, B and Fig. S3; left columns). Interestingly, in some infiltrates, cells displaying a lower GFP fluorescence intensity were detected which exhibited a much smaller cell size than GFP-expressing AGECs. Furthermore, their morphology was comparable to the surrounding cells of the infiltrate, suggesting that these cells were not directly infected cells but might rather be phagocytic cells which had taken up remnants of MCMV infected cells including GFP (Fig.4, middle column).

It was recently shown that IFNγ-secreting CD4 T cells are necessary for the migration of herpes simplex virus-specific CD8 T cells to the site of infection [24]. However, during MCMV infection, migration of CD8 T cells, B cells as well as NK cells, CD11c and CD11b expressing cells to infected cells of the SG was not impaired in the absence of CD4 T cells (Fig. 4C and Figs. S3 and S4). Distribution of these cells in the SG examined by immunohistological analysis were comparable between MCMV-infected MHCII^-/-^ and B6 mice with the only exception that the former strain had increased numbers of infected foci (Fig. 4C and Fig. S3).

### MHC class I and II expression is only detectable on very few infected cells of the SG and only at very low levels

**Figure 5:**
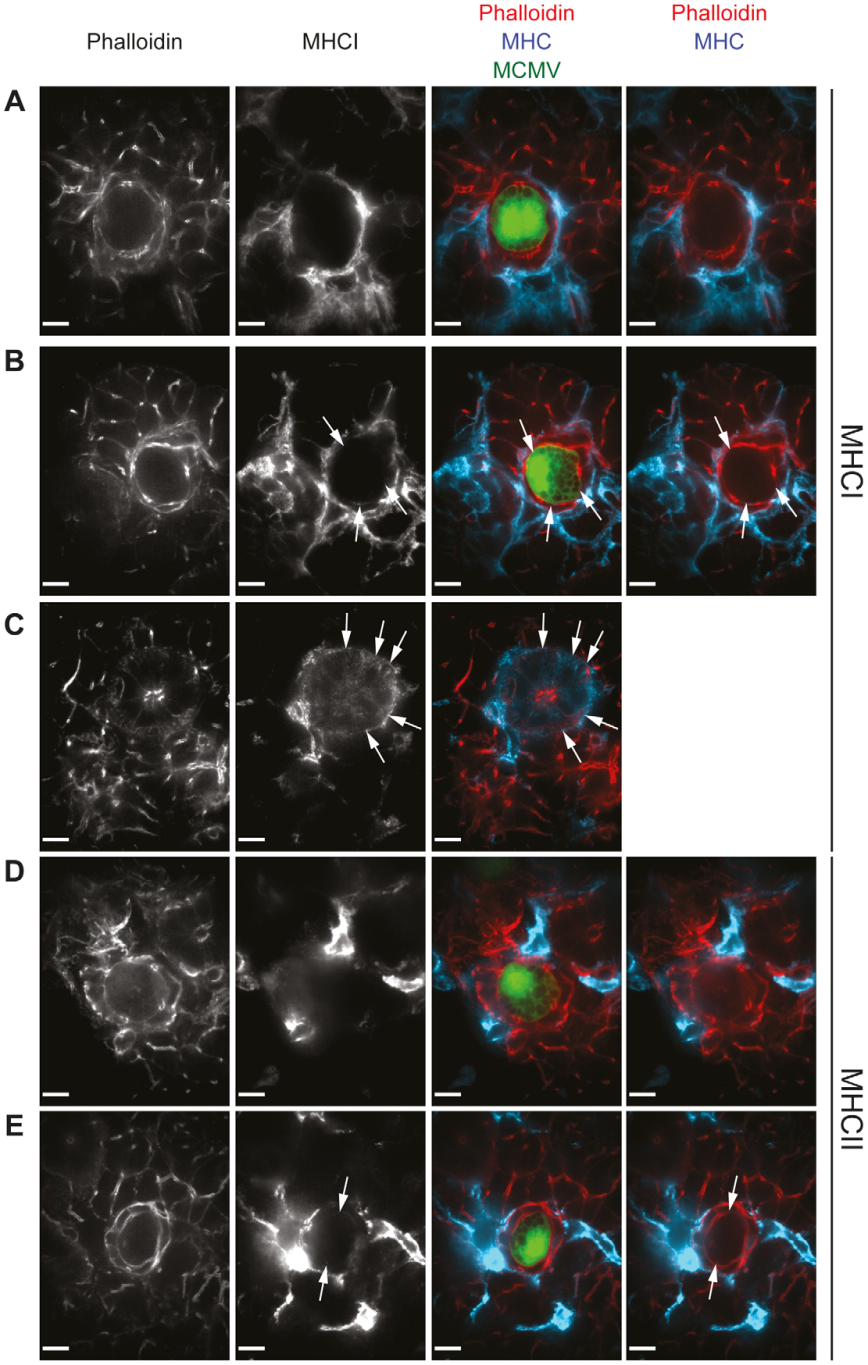
MHC class I and II expression is absent in intact MCMV infected cells and may be re-expressed at low levels on infected cells in proximity to leukocyte infiltrates. Three weeks post MCMV-GFP infection, SG sections isolated from B6 mice were stained for MHC class I (A to C; blue) or MHC class II (D and E; blue) as well as with phalloidin (A to E; red), visualizing actin. MCMV-infected cells which are situated distal (A and D) or in proximity (B and E) to cell infiltrates were analyzed. Expression of MHC class I on uninfected AGECs is shown in C. Arrows point out weak MHC class I or MHC class II expression on AGECs. Confocal images were taken with 100 times magnification. Scale bar indicates 10 µm. One representative picture of a minimum of 10 is shown.

We hypothesized that CD8 T cells, in contrast to CD4 T cells, are unable to control viral replication in the SG because they are not exposed to their cognate antigen in this particular organ. MCMV suppresses not only MHC class I but also MHC class II expression on infected cells [25], [26], [27], [28] and inhibition of MHC class I expression might be more pronounced in AGECs than MHC class II expression. To test this hypothesis, sections of SG were stained for MHC class I and II expression three weeks post MCMV-GFP infection and analyzed by confocal microscopy. Neither MHC class I nor MHC class II molecules were detectable on directly infected GFP-positive cells when distal to cell infiltrates (Fig. 5A and D). However, once proximate to leukocyte infiltrates, very few infected cells expressed very low levels of MHC class I and class II on their cell surface, but to a much lower extent than on the close-by leukocyte infiltrate (Fig. 5B and E). Overall, 86% of all GFP-infected cells had no detectable surface expression of MHC class I and only 14% expressed MHC class I at very low levels. Comparably, only 14% of GFP-expressing cells in the SG were slightly positive for MHC class II on their cell surface. Low or absent MHC expression on directly infected cells stands in line with published data showing that MCMV very potently inhibits antigen presentation via down-regulation of MHC molecules *in vitro* [25], [26], [27], [28], [29]. MHC class I expression on uninfected AGECs was concentrated to the apical side of the cells and was overall low in comparison to leukocytes (Fig. 5C). MHC class II expression was completely absent in uninfected AGECs (data not shown). The comparably rare and low expression patterns of MHC class I and II by infected AGECs argues against the hypothesis that CD4 T cells would preferentially interact directly with MCMV-infected AGECs.

### SG resident APCs are deficient in cross-presentation

Although directly infected cells of the SG lacked MHC class I and II expression, other cells present in close proximity expressed high levels of both these molecules (Fig. 5). It is likely that these non-infected bystander APCs are presenting MCMV-derived antigens to MCMV-specific T cells. As these bystander cells were clearly not directly infected since they lacked GFP expression, these cells would have to present MCMV antigens from an exogenous source. It is conceivable that MHC class II-restricted MCMV epitopes might be presented on these APCs much more efficiently than MHC class I-restricted epitopes. In the spleen, cross-presentation of exogenous antigens on MHC class I molecules is largely restricted to a distinct dendritic cell (DC) subset, identified by CD11c, MHCII, CD8α, DEC205 expression and concomitant absence of B220 and CD4 expression [30].

**Figure 6:**
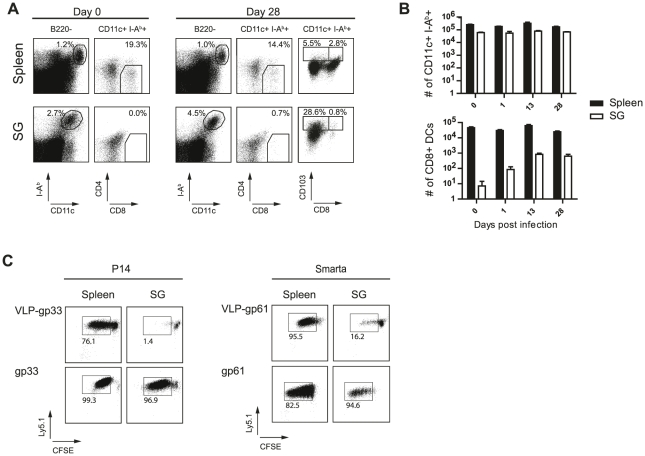
Cross-presenting APCs are absent in SG tissue. (A) Spleen cells and SG resident leukocytes from naïve or 4 week MCMV infected mice were stained for I-A^b^, CD11c, CD8α, CD4 or CD103 and B220. Left plots are gated on B220 negative cells and stained for I-A^b^ and CD11c, right plots are gated on B220^−^, MHC class II^+^, CD11c^+^ cells and stained for CD4 respectively CD103 and CD8α. Frequencies of gated cells among the parent population are indicated. B) Total numbers of total CD11^+^ I-A^b+^ B220^−^ (upper diagram) or CD11^+^ I-A^b+^ B220^−^ CD8α^+^ CD4^−^ (lower diagram) DCs resident in the spleen (black bar) or APCs in the SG (open bar) are shown for different time points post MCMV infection. C) DCs were isolated four weeks post MCMV infection from spleen (left column) or APCs from the SG (right column) and pulsed with VLPs cross-linked to either the LCMV-derived gp33 (upper left plots) or to gp61 (upper right plots) peptides or pulsed with the peptide (lower plots). 10^5^ naïve CFSE-labeled gp33-specific CD8 T cells (P14) or naïve CFSE-labeled gp61-specific CD4 T cells (Smarta) were added to equal numbers of splenic DCs or SG derived APCs. After 5 days of incubation, CFSE dilution was analyzed by gating on P14 or Smarta cells. One representative example of three individual experiments is shown.

To test if SG-resident APCs are phenotypically related to cross-presenting splenic DCs, splenic DCs as well as SG-derived APCs were stained for surface expression of CD11c, MHC class II, B220, DEC205, CD4, and CD8α at different time points post infection. CD11c^+^, MHCII^+^, CD8α^+^, DEC205^+^, B220^−^, CD4^−^ cross-presenting DCs were detectable in the spleen of B6 mice at all the time points examined (Fig. 6A and B). In contrast, this DC subset was almost completely absent in the SG isolated from naïve animals (Fig. 6A). Even when the SG tissue was heavily infected by MCMV two and four weeks post infection, frequencies of CD11c^+^, MHCII^+^, CD8α^+^, DEC205^+^, B220^−^, CD4^−^ cells among the overall SG-resident APC population remained very low (<1.5%) and total numbers remained far below their splenic counterparts. In peripheral organs such as the gastrointestinal tract, skin and the lung CD103^+^ DCs represent one of the major DC subset able to cross-present exogenous antigen to CD8 T cells [31], [32] and on day 28 30% of the SG-resident CD11c^+^ I-A^b+^ APCs expressed CD103 on their cell surface (Fig. 6 A).

To formally assess the capacity of SG-resident APCs to process and present exogenous antigen on MHC class I or class II molecules, we isolated SG-resident APCs and splenic DCs. APCs were left untreated or were pulsed with virus like particles (VLPs) linked either to the LCMV-derived MHC class I-restricted epitope gp33 or the MHC class II-restricted gp61 epitope. As controls, APCs were pulsed directly with the respective peptides. We used VLPs to assess cross-presentation capacities of splenic DCs and SG-derived APCs as they represent a well-characterized particulate antigen which has been previously shown to depend on cross-presentation for MHC class I antigen loading using splenic DCs [33]. Identical numbers of spleen- or SG-derived APCs were then added to naïve CFSE-labeled TCR transgenic CD8 T cells recognizing the gp33 epitope (P14 cells) or TCR transgenic CD4 T cells specific for the gp61 epitope (Smarta cells). P14 cells recognized their antigen on splenic DCs either pulsed with VLP-gp33 or their cognate peptide (Fig. 6C). However, salivary gland-derived APCs were not able to cross-present the cognate peptide to P14 T cells when pulsed with VLP-gp33. SG-derived APCs were not per se unable to stimulate CD8 T cells as they were able to do so when pulsed with the gp33 peptide. In contrast, Smarta cells proliferated extensively when incubated with either SG-derived APCs or splenic DCs pulsed with VLP-gp61. These results demonstrate, on a functional level, that SG-resident APCs are not able to cross-present exogenous antigens to CD8 T cells. As we could never detect GFP^+^ CD11c^+^ cells in any of the SG sections analyzed, we conclude that they are not directly infected by MCMV and hence do not present endogenous MCMV-derived antigens on MHC class I molecules. However, in line with the notion that SG-resident APCs are responsible for MCMV antigen presentation, we were able to detect in some rare cases CD11c^+^ MHC class I (Fig. S5A) or MHC class II (Fig, S5B) expressing cells with focal GFP inclusions which most likely represent APCs which had phagocytosed remnants of infected AGECs. Based on our functional *in vitro* data, we propose that these might be the very cells being responsible for local MCMV-derived antigen presentation to CD4 T cells.

### MCMV lacking MHC class I immune evasion genes is controlled in the SG in absence of CD4 T cells

**Figure 7:**
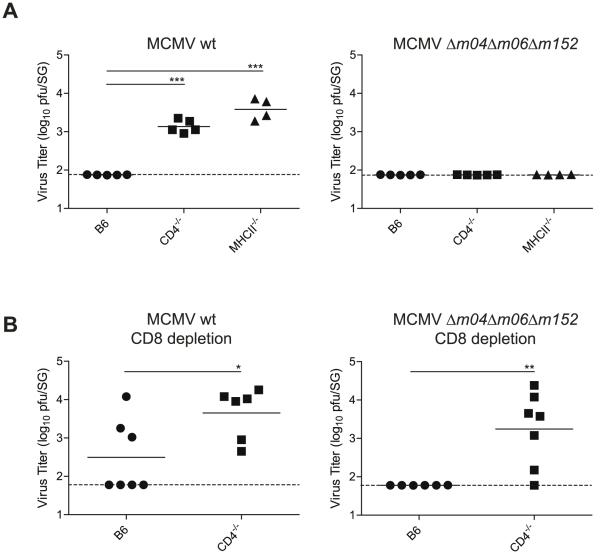
MCMV control in the SG in presence or absence of viral immune evasion genes. (A) B6, CD4^-/-^ or MHCII^-/-^ mice were infected with MCMV-*Δm04Δm06Δm152* or its parental BAC-derived virus (MCMV wt) and SG virus titers were determined 28 days post infection. (B) B6 and CD4^-/-^ mice were depleted of CD8 T cells and infected with MCMV-*Δm04Δm06Δm152* or its parental BAC-derived virus (MCMV wt) and SG virus titers were determined 28 days post infection. Combined data of two independent experiments is shown. (* p<0.05, ** p<0.01, *** p<0.001, 2-tailed unpaired student's t-test).

To directly assess whether the absent MHC class I expression on infected AGECs due to MCMV-encoded immune evasion genes was responsible for the exclusive CD4 T cell-mediated control of MCMV in the SG, we made use of a triple MCMV mutant lacking all three major MHC class I immune evasion genes *m04*, *m06* and *m152 (Δm04Δm06Δm152*) [34]. B6, CD4^-/-^ and MHCII^-/-^ mice were infected with *Δm04Δm06Δm152* MCMV and its parental BAC-derived virus and viral titers were examined 28 days post infection in the SG. While MHCII^-/-^ and CD4^-/-^ mice were unable to control wild type (WT) MCMV replication, they were able to control *Δm04Δm06Δm152* MCMV by day 28 post infection (Fig. 7A), indicating that the strict requirement for CD4 T cells to control SG MCMV infection no longer holds in absence of MCMV-encoded MHC class I immune evasion genes. Consistent with the notion that CD8 T cells become major contributors for control of SG MCMV infection in absence of MHC class I immune evasion genes, CD8 T cell depletion resulted in significantly impaired control of *Δm04Δm06Δm152* MCMV replication in the absence of CD4 T cells (Fig. 7B).

## Discussion

In this study we unravel the underlying mechanisms of the unique requirement for CD4 T cells to control MCMV replication in the SG. We show that CD4 T cells control MCMV replication in the SG by exerting direct antiviral effector functions via IFNγ secretion and not by providing helper functions to CD8 T cells or B cells. While robust CD8 T cell responses were detected in presence or absence of CD4 T cells in the SG, they were unable to control MCMV replication due to the exquisite efficiency of MCMV to downregulate MHC class I expression on infected AGECs. In absence of MCMV-encoded MHC class I immune evasion genes, CD8 T cells gained ability to control SG MCMV infection also in absence of CD4 T cells.

However, in the absence of antiviral active CD4 T cells, B cells and antibodies can contribute to control viral replication in the SG on the long term, as infectious virus was eventually cleared in CD4^-/-^ mice in all organs examined as opposed to MHCII^-/-^ mice. One major difference between MHCII^-/-^ and CD4^-/-^ mice is that the latter strain is able to mount isotype-switched antibody responses [35], [36]. MCMV-binding IgG and neutralizing antibodies were indeed detectable in MCMV-infected CD4^-/-^ mice, but were completely absent in MHCII^-/-^ animals (Fig. S6 and ). As MCMV-specific antibodies were previously shown to inhibit viral dissemination during MCMV infection [12], [37], they are likely to be responsible for late differences in MCMV control observed between MHCII^-/-^ and CD4^-/-^ mice.

*In vitro*, IFNγ and TNFα can exert strong and synergistic inhibition of MCMV replication in fibroblasts [38]. Systemic *in vivo* neutralization of IFNγ and TNFα in CD8 T cell depleted MCMV infected BALB/c mice or in MCMV-infected lethally irradiated mice transferred with MCMV-primed splenocytes resulted in decreased MCMV control [6], [7]. Thus, CD4 T cells were suggested to act via secretion of IFNγ and TNFα to limit viral replication in the SG. However, in these studies, unlike ours, there was no formal proof that CD4 T cells themselves and no other cells secrete those cytokines. We were able to show for the first time that CD4 T cells exert direct antiviral mechanisms by producing IFNγ and to a much lower extent - if at all - via TNFα production or perforin-mediated effector mechanisms. At this point we cannot formally exclude that CD4 T cells might interact with other cells which then produce TNFα, thereby leading to control of viral replication in the SG. However, our observation that lytic MCMV replication is not controlled in chimeric mice which specifically lack IFNγR expression on non-hematopoietic cells would rather argue against a pivotal role of additional effectors including TNFα.

It was proposed that NK-like cells might synergize with CD4 T cells to control viral replication in the SG [4], due to the fact that mice depleted of NK cells by anti-NK1.1 or anti-asialoGM1 antibodies showed increased viral titers in the SG [11], [39], [40], [41]. Our data does not exclude a synergism between CD4 T and NK cells, we can only conclude that a potential NK-CD4 T cell interaction is unlikely to be mediated via IFNγ. Further, the NK cell compartment in the SG was not affected by the absence of CD4 T cells: NK cells were present in the SG of CD4 T cell deficient animals, absolute numbers were comparable to wild type mice and we were unable to detect any differences in NK cell function between MHCII^-/-^ and B6 mice (Fig. S7 and Text S1). Furthermore, SG-resident NK cells were recently shown to have functional deficits and peripheral NK cells appear to be poorly recruited to the SG during MCMV infection [42]. Finally, in contrast to many reports demonstrating a role for NK cells in limiting early MCMV replication [11], [39], [40], [41], [43], a recent study showed a negative impact of NK cells on MCMV control due to curtailed MCMV-specific T cell responses [44].

Why then is IFNγ secreted by CD4 T cells so crucial to control MCMV in the SG whereas IFNγ production by other immune cells such as CD8 T cell or NK cells is insufficient? Different reasons could account for this: a) other immune cells might not produce IFNγ in the SG; b) CD4 T cells but no other IFNγ-producing cells migrate into close proximity of infected cells; c) CD4 T cells might enable other immune cells to migrate to infected cells; d) CD4 T cells, in contrast to CD8 T cells, might recognize directly infected cells; e) antigens might not be presented directly on infected cells but on bystander APCs that are unable to cross-present and hence to activate CD8 T cells.

We show here evidence for the last scenario as both MCMV-specific CD8 as well as CD4 T cells from the SG were able to secrete IFNγ, total numbers of IFNγ^+^ CD8 T cells exceeded by far total numbers of IFNγ^+^ CD4 T cells, CD8 T cells were localized at the same sites as CD4 T cells in the SG, the cellular composition of the infiltrates were not changed in absence of CD4 T cells, most directly infected cells in the SG were MHC negative and SG-resident APCs were capable of processing and presenting particulate antigen on MHC class II but not MHC class I molecules. Importantly, we have no evidence for direct infection of SG APCs, at least at the time points analyzed (Fig. 5 and data not shown). Furthermore, we found some MHC class I and II positive CD11c^+^ cells within densely populated leukocyte infiltrates in the SG which displayed discrete inclusion of cargo derived from MCMV-infected cells, suggestive of phagocytes which had taken up remnants of MCMV-infected AGECs.

Our observations that we only rarely detected MHC class I and II molecules on directly infected AGECs stands in line with findings that MCMV down-regulates very efficiently MHC molecules, albeit this has mostly been shown *in vitro* for infected fibroblasts, macrophages and dendritic cells [25], [26], [27], [28], [29]. In fact our study demonstrates for the first time that MCMV can indeed suppress surface expression of MHC complexes on AGECs *in vivo* as well. MCMV expresses several gene products, so called immune-evasins, that impair antigen presentation on MHC class I molecules (reviewed by [29]). Interestingly, genetic deletion of these immune-evasins resulted in increased MCMV immune control specifically in the SG in a CD8 T cell dependent manner in BALB/c mice [25], suggesting that MCMV expressed immune-evasins are particularly potent in AGECs compared to cells of other tissues. We corroborated and extended this observation by showing that CD4 T cells were dispensable for MCMV control in the SG in the absence of the virus-encoded *m04*, *m06* and *m152* immune-evasins. Up to date we were not able to study MHC class I expression on AGECs infected with *Δm04Δm06Δm152* MCMV as the mutant strain currently available does not express GFP. In future experiments we will generate a GFP expressing *Δm04Δm06Δm152* MCMV strain to address this open question.

**Figure 8:**
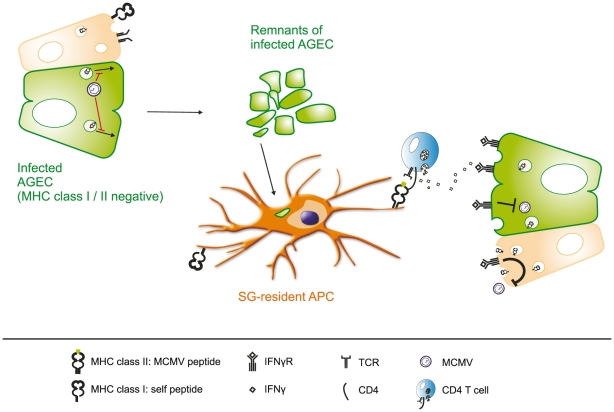
Virus-specific CD4 but not CD8 T cells control MCMV replication in the salivary gland. Surface expression of MHC class I and II is effectively suppressed in infected acinar glandular epithelial cells (AGECs), thereby inhibiting direct recognition by MCMV-specific CD8 and CD4 T cells. Phagocytic APCs situated in close proximity to virus-infected cells take up MCMV antigens, either in from of defective viral particles or apoptotic bodies of infected cells, and present MCMV-derived peptide antigens on MHC class II molecules to CD4 T cells but not to CD8 T cells due to their inability to cross-present particulate antigens. Activated MCMV-specific CD4 T cells secrete IFNγ which consequently binds to its receptor on the cell surface of the infected cell as well as on adjacent AGECs. Activation of IFNγR signaling cascade on adjacent cells induces an antiviral state, rendering them resistant to MCMV replication and IFNγ signaling in infected cells leads to the termination of MCMV replication.

In summary, the results of our study strongly support a model in which virus-specific CD4 T cells but not CD8 T cells control viral replication in the SG, thereby eventually curtailing horizontal transmission (Fig. 8): In infected AGECs MCMV immune-evasins inhibit trafficking of MHC class I molecules to the cell surface and shut down the induction of MHC class II expression. MCMV antigens released by the infected cells or remnants of infected cells are taken up by local APCs which are unable to cross-present particulate antigens but able to process and present on MHC class II, thereby selectively inducing MCMV-specific CD4 T cells to secrete IFNγ. IFNγ binds to its receptors present on infected cells or / and on adjacent cells and exerts its antiviral function by interfering with viral replication or rendering cells resistant for subsequent MCMV infection.

## Material and Methods

### Ethics statement

This study was carried out in in strict accordance to the guidelines of the animal experimentation law (SR 455.163; TVV) of the Swiss Federal Government. The protocol was approved by Cantonal Veterinary Office of the canton of Zurich, Switzerland (Permit number 145/2008). All surgery was performed under isoflurane anesthesia and animals were treated pre- and post-surgically from day-1 to day+7 with the analgesic Buprenorphine. All efforts were made to minimize suffering.

### Mice, *in vivo* T cell depletion and peptides

C57BL/6, MHCII^-/-^ [45], CD4^-/-^ [46], CD8^-/-^, B6.129P-*Igh-J^tm1Cgn^* (JHT) [47], IFNγ^-/-^ (Charles River Laboratories), TNFα^-/-^ (B6.129-*TNF* ^tm1Ljo^, [48]), PKOB [49] and IFNγR-deficient mice were kept under specific pathogen free (SPF) conditions and were infected intravenously with 10^6^ plaque forming units (PFU) of MCMV-*Δm157*, MCMV wt, MCMV*-Δ04Δ06Δ152*, MCMV*-Δm157*-luciferase, or 5×10^5^ PFU MCMV-*Δm157*-GFP between 6 and 12 weeks of age. For CD8 T cell depletion, mice were injected i.p. with 0.2 mg of purified anti-mouse CD8 monoclonal antibody (YTS 169.4, [50]) 3 and 1 days before infection and then weekly.

The MCMV-derived M45_aa985-993_ and M38_aa316-323_ as well as the LCMV-derived gp33 and gp61 peptides were purchased from NeoMPS (Strasbourg, France). Production of a crude lysate of MCMV infected cells was previously described [8]. Production and purification of VLP-gp33 (HBcAg-p33) and VLP-gp61 (HBcAg-p33) was previously described [51].

### Generation and characterization of bone marrow chimeras

CD4^-/-^ mice were irradiated (950 rad γ) and reconstituted with a 1∶1 mixture of bone marrow from CD4^-/-^ and CD4^-/-^ or IFNγ^-/-^ or TNFα^-/-^ or PKOB or C57BL/6 mice. In other experiments, irradiated IFNγR^-/-^ or C57BL/6 recipients were reconstituted with bone marrow of IFNγR^-/-^ or C57BL/6 mice.

### Viruses

Generation of the recombinant MCMV-*Δm157* (lacking the *m157* gene of MCMV) was previously described [8]. The *m157*-deficient MCMV mutant expressing the luciferase gene was a kind gift of Dr. M. Mach (Erlangen, Germany) [16]. The MCMV mutant expressing green fluorescent protein (GFP) under the *m157* promoter was described before [52] as well as MCMV-*Δm04Δm06Δm152* [34]. MCMV was propagated on mouse embryonic fibroblasts (MEFs) and viral titers were determined using plaque forming assays as described in [53].

### In vivo bioluminescence imaging

Shaved mice were injected intravenously with 0.5 mg D-luciferin in 200 µL PBS and immediately anaesthetized using isoflurane. Ten minutes after luciferin injection, bioluminescence was recorded over a 300-second integration period by a cooled CCD camera system (IVIS Imaging System 200 Series, Caliper Life Sciences AG, Oftringen, Switzerland). Anesthesia was maintained during imaging by nose cone delivery of the anesthetic. Relative intensities of transmitted light from the in vivo bioluminescence were represented as pseudocolor imaging.

### Antibodies

PE-conjugated peptide-MHC class I tetrameric complexes were generated as previously described [54]. The following monoclonal antibodies were either purchased from BD Pharmingen (Allschwil, Switzerland) or from BioLegend (Lucerna Chem AG, Luzern, Switzerland) and used for stainings: anti-CD8 (FITC, PerCP, APC, PacificBlue, APC-Cy7), anti-CD4 (PE, PerCP, PacificBlue), anti-IFNγ (APC), anti-B220 (PE-Cy7), anti-Ly6C (FITC), CD11b (PerCP), anti-CD11c (APC), anti-I-A^b^ (PE).

### Cell stimulation, immunofluorescent staining and analysis

Lymphocytes were isolated from spleen, lung, liver and SG as previously described [55]. Cells were surface stained with directly labeled Abs or peptide-MHC class I tetramer complexes followed by erythrocyte lysis. For intracellular cytokine stainings, CD8 T cells were stimulated with 1 µg/ml peptide and CD4 T cells with 10 µg/ml cell lysate in the presence of 10 µg/ml brefeldin A (Sigma-Aldrich) at 37°C for 6 h. Cell surface staining was done as described above, followed by fixation and permeabilization using 500 µl of FIX/Perm solution (FACSLyse diluted to 2x concentration and 0.05% Tween 20) for 10 min at room temperature. Cells were washed and stained with directly labeled Abs against IFNγ and TNFα. Multiparameter flow cytometric analysis was performed using a FACS LSRII flow cytometer (BD, Allschwil, Switzerland) with FACS DIVA software (BD, Allschwil, Switzerland). List mode data were analyzed using FlowJo software (Treestar, San Carlos, CA).

### Immunofluorescence microscopy

SG were isolated from infected animals, fixed for 1 h in PBS containing 4% PFA at 4°C and incubated overnight in PBS containing 20% sucrose, followed by tissue embedding in O.C.T. compound (Sakura, Torrance, CA), snap-freezing in liquid N_2_ and storing at −80°C. Cryosections of 16 µm thickness were prepared, completely air-dried for 2 to 6 hours before recovery by a brief rinse in PBS. Sections were blocked (PBS with 10% FCS) for one hour. Primary antibody (CD4 (clone YTS191.1), CD8 (clone YTS156), CD11c (BD Pharmingen), I-A^b^ (BioLegend), MHC class I (Santa Cruz)) was added in staining buffer (1% mouse serum in PBS) for one hour before extensive washing with PBS. Incubation with secondary antibodies (anti-hamster IgG (Cy3/Cy5), Phalloidin (647-labeled Fluoprobes), anti-rat IgG (Cy3/ Cy5), all purchased from Sigma-Aldrich) containing DAPI (Sigma-Aldrich) diluted in staining buffer lasted one hour, followed by extensive washing and fixation with 1% PFA and mounting with VectaShield (Vector Laboratories, Burlingame, CA). Samples were viewed and analyzed with an inverted confocal microscope (Axiovert 200, Carl Zeiss, Inc.), equipped with an oil-phase contrast objective (Plan Neofluar, Carl Zeiss, Inc.), an ultraview confocal head (PerkinElmer, location) and a krypton argon laser (643-RYB-A01, Melles Griot). Data analysis was done with Velocity (Improvision).

### Cross-presentation assay

One month post MCMV infection four to five spleens and SG were digested using Liberase TL Research Grade (Roche, Rotkreuz, Switzerland) and DNaseI according to manufacturer's instruction, except that SG tissue was dissociated previous to digestion and in between two incubation periods of 20 minutes. DCs from the spleen and APCs from the SG were purified with CD11c MACS beads according to manufacturer's instruction, pulsed with either 1 µg/ ml VLPs or 10^−8^ M peptide for 1 hour at 37°C. After intensive washing, APCs were counted and purity was determined by FACS analysis. Equal numbers of splenic DCs and SG APCs (varying inter experimentally between 0.5 to 1×10^5^ cells) were added to 10^5^ TCR transgenic CD8 or CD4 T cells previously labeled with CFSE (Invitrogen, Basel, Switzerland; end concentration 2 mM) and incubated for 5 days. CD8 and CD4 T cells were MACS-purified from spleens of Ly5.1^+^ P14 or Ly5.1^+^ Smarta transgenic mice, respectively. 5 days later cells were labeled with antibodies against CD4, CD8 and Ly5.1 and analyzed by FACS.

## Supporting Information

Figure S1 **Virus titer kinetics in B6, CD4^-/-^, MHCII^-/-^ and JHT mice.** B6, CD4^-/-^, MHCII^-/-^ or JHT mice were infected with MCMV and viral titers were determined by plaque assay up to 120 days post infection in spleen, liver, kidney and SG. One of three independent experiments is shown.
(EPS)

Figure S2 **Helper functions exerted by CD4 T cells are dispensable for MCMV control.** A) 4 weeks and B) 8 weeks post infection viral titers in the SG were compared between CD8^-/-^, JHT, MHCII^-/-^ and wild type mice (B6). Statistical analysis was done using unpaired t-test. n.s. not significant, * p<0.05
(EPS)

Figure S3 **B cells, CD11b^+^ and NK cells infiltrate the infected SG tissue independent of CD4 T cells.** SGs were isolated three weeks post infection from B6 (left two columns) or MHCII-/- (right two columns) mice infected with a GFP-expressing MCMV mutant. Cryosections of SGs were counterstained with either anti-B220 (A; red) to detect B cells, anti-CD11b (B; red), anti-asialoGM1 (C; red) to detect NK cells and CD11c (A to C; blue). MCMV-bearing AGECs (green) situated distal to immune infiltrates (first and third column) and immune infiltrates (second and fourth column) are displayed. Confocal images were taken with 20 times magnification. Scale bar indicates 100 µm. One representative picture of a minimum of 10 is shown.
(TIF)

Figure S4 **Composition of cellular infiltrate in the SG of infected mice.** B6, CD4^-/-^ or MHCII^-/-^ mice were infected with MCMV. 28 days post infection leukocytes were isolated from the SG and pooled in between experimental groups. Total numbers of lymphocytes, CD8 T cells, CD4 T cells, B cell, NK cells (CD49^+^, TCRβ^−^), NKT cells (CD49^+^, TCRβ^+^), CD11c^+^ cells and CD11b^+^ cells were determined by flow cytometry.
(EPS)

Figure S5 **MHC class I and II expression on CD11c^+^ cells with focal GFP inclusions.** Three weeks post MCMV-GFP infection, SG sections isolated from B6 mice were stained for MHC class I (A; blue) or MHC class II (B; blue) molecules as well as with phalloidin (A and B; red), visualizing actin. Few CD11c^+^ cells with focal GFP inclusions (arrows) were found which expressed MHC class I (A) or MHC class II (B). Confocal images were taken with 40 times magnification. Scale bar indicates 10 µm. One representative picture of minimum 3 is shown.
(TIF)

Figure S6 **MCMV-specific antibody response.** B6, CD4^-/-^, MHCII^-/-^ or JHT mice were infected with MCMV and MCMV-specific antibodies were longitudinally determined by ELISA (A) or by neutralization assay (B). Titers refer to 50% neutralization capacity. One of three independent experiments is shown.
(EPS)

Figure S7 **NK cell frequencies, activation and function in presence or absence of CD4 T cells.** B6 and MHCII^-/-^ mice were infected with MCMV and frequencies of NK cells were determined in the spleen and SG at the indicated time points post infection by flow cytometry. IFNγ production within NK cells was assessed directly *ex vivo* as well as the percentage of activated NK cells (identified by CD69 expression). One of two independent experiments is shown.
(EPS)

Text S1 **Supporting information.**
(DOC)
