# Supplementary figures and images for: Absence of Cross-Presenting Cells in the Salivary Gland and Viral Immune Evasion Confine Cytomegalovirus Immune Control to Effector CD4 T Cells
Source: PLoS Pathog. 2011 Aug 25;7(8):e1002214. doi: 10.1371/journal.ppat.1002214 (PMC3161985; doi:10.1371/journal.ppat.1002214)

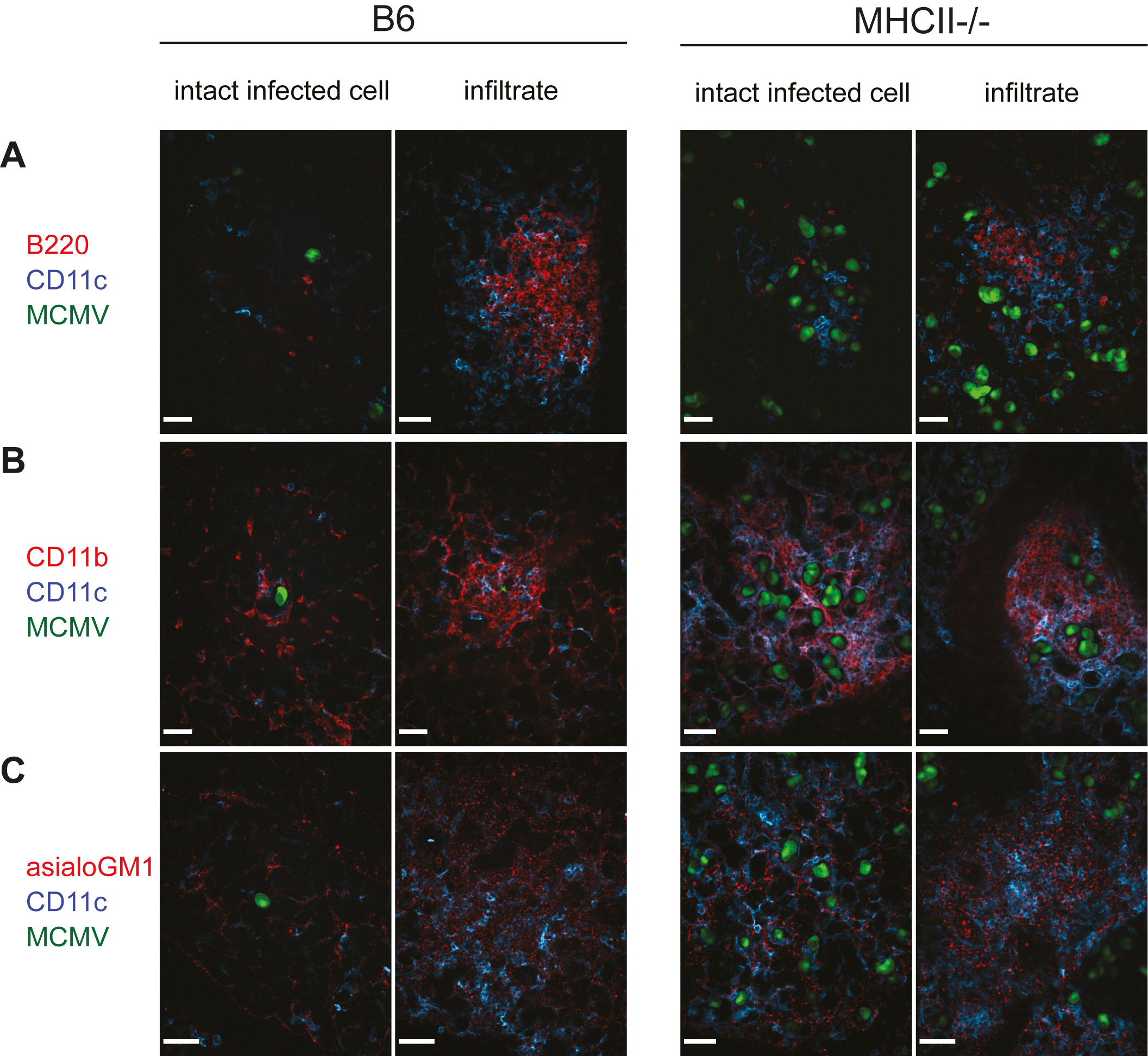

Supplement: Figure S3 — B cells, CD11b+ and NK cells infiltrate the infected SG tissue independent of CD4 T cells. SGs were isolated three weeks post infection from B6 (left two columns) or MHCII-/- (right two columns) mice infected with a GFP-expressing MCMV mutant. Cryosections of SGs were counterstained with either anti-B220 (A; red) to detect B cells, anti-CD11b (B; red), anti-asialoGM1 (C; red) to detect NK cells and CD11c (A to C; blue). MCMV-bearing AGECs (green) situated distal to immune infiltrates (first and third column) and immune infiltrates (second and fourth column) are displayed. Confocal images were taken with 20 times magnification. Scale bar indicates 100 µm. One representative picture of a minimum of 10 is shown. (TIF) [file ppat.1002214.s003.tif]

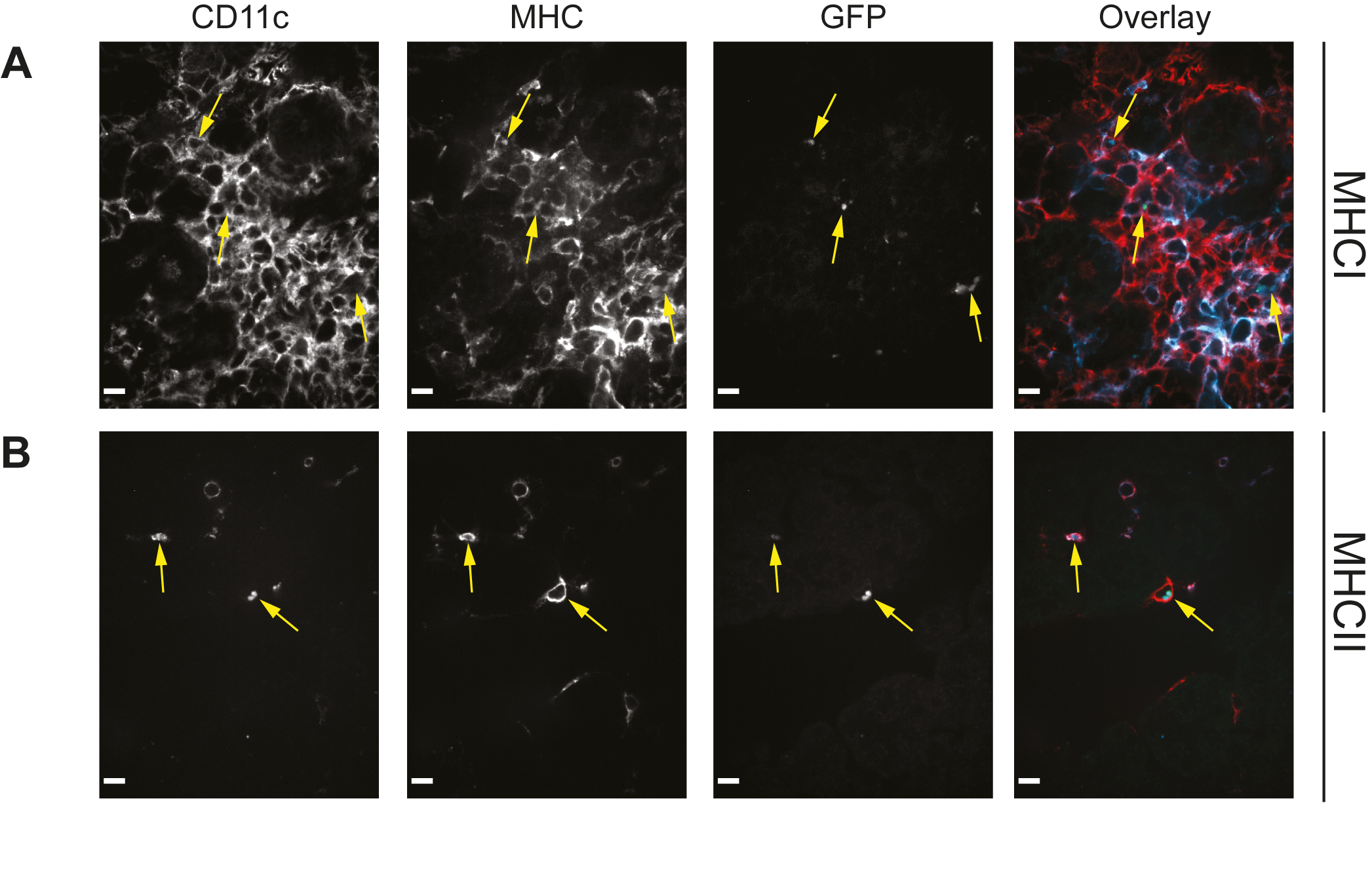

Supplement: Figure S5 — MHC class I and II expression on CD11c+ cells with focal GFP inclusions. Three weeks post MCMV-GFP infection, SG sections isolated from B6 mice were stained for MHC class I (A; blue) or MHC class II (B; blue) molecules as well as with phalloidin (A and B; red), visualizing actin. Few CD11c+ cells with focal GFP inclusions (arrows) were found which expressed MHC class I (A) or MHC class II (B). Confocal images were taken with 40 times magnification. Scale bar indicates 10 µm. One representative picture of minimum 3 is shown. (TIF) [file ppat.1002214.s005.tif]
