# Supplementary material for: Absence of Cross-Presenting Cells in the Salivary Gland and Viral Immune Evasion Confine Cytomegalovirus Immune Control to Effector CD4 T Cells
Source: PLoS Pathog. 2011 Aug 25;7(8):e1002214. doi: 10.1371/journal.ppat.1002214 (PMC3161985; doi:10.1371/journal.ppat.1002214)
Supplement: Text S1 — Supporting information. (DOC) [file ppat.1002214.s008.doc]

**Text S1. Supporting information.**

**ELISA**

ELISA plates were coated with 10µg crude MCMV lysate (prepared from MCMV infected mouse embryonic fibroblasts (MEFs) as described in Walton et al. in 1ml 0.1M NaHCO_3_ (pH 9.6) overnight at 4°C. After extensive washing, plates were blocked with 1% BSA in PBS for 2 hours at room temperature. Sera of naïve and MCMV infected mice were diluted 1:40 in 0.1 % BSA in PBS and added to the ELISA plates for 1 hour at room temperature. MCMV-specific IgG antibodies were detected with an HRPO coupled anti-mouse IgG-specific antibody (1:1000 in 0.1% BSA in PBS; Sigma-Aldrich Chemie, Buchs, Switzerland), followed by addition of ABTS/ H_2_O_2_ solution containing 0.2 mg/ml 2,20-azino-di-(3 ethylbenzthiazoline sulfonic acid), 0.1 M NaH2PO4, 0.04% H2O2, pH 4. Plates were read at 405 nm in a Victor3 reader (Wallac 1420, PerkinElmer, Waltham, MA,USA).

**MCMV neutralization assay**

Serial dilutions of sera were incubated with 1000 pfu MCMV *Δ157luc* for 1 hour at 37^o^ C. The mixture was added to 3T3 fibroblasts in 24-well plates, centrifuged 30 min at 1200 g and incubated for 4 hours at 37^o^ C. Culture medium was exchanged and infection continued for 24 hours. Thereafter, cells were lysed in 100 µl lysis buffer and 50 µl was used to measure luciferase activity. The neutralization titer of a serum was expressed as the reciprocal of the highest serum dilution that reduced virus load as determined from control infections by 50 %.

**IFNγ-secretion by NK cells**

To measure direct *ex vivo* IFNγ production by NK cells, mice were injected with 250µg Brefeldin A i.v. 4 hours prior to analysis. Lymphocytes were isolated in Brefeldin A containing medium and incubated for another 4 hours at 37°C. Intracellular cytokine staining was performed as described for T cells.
